# Integrating computational insights in gold nanoparticle-mediated drug delivery: enhancing efficacy and precision

**DOI:** 10.3389/fmedt.2025.1528826

**Published:** 2025-02-24

**Authors:** Amnah Alalmaie, Huda Turki Alshahrani, Manar Alqahtani, Zeyad Alshahrani, Shahad Alahmari, Asilah Asiri, Bandar Alqadi, Abdulrahman Alshahrani, Safar Alshahrani, Md Habban Akhter

**Affiliations:** ^1^ Department of Pharmaceutics, College of Pharmacy, King Khalid University, Abha, Saudi Arabia; ^2^ Faculty of Pharmacy, DIT University, Dehradun, India

**Keywords:** gold nanoparticles (AuNPs), drug delivery systems, targeted cancer therapy, computational, artificial ntelligence in nanomedicine

## Abstract

Gold nanoparticles (AuNPs) have emerged as a versatile platform in biomedical applications, particularly in drug delivery, cancer therapy, and diagnostics, due to their unique physicochemical properties. This review focuses on the integration of computational methods and artificial intelligence (AI) with nanotechnology to optimize AuNP-based therapies. Computational modeling is essential for understanding the interactions between AuNPs and biological molecules, guiding nanoparticle design for improved targeting, stability, and therapeutic efficacy. Recent advancements, including AI-driven models in precision cancer therapy and the combination of AuNPs with antimicrobial peptides (AMPs) to combat drug-resistant pathogens, are highlighted. The review also discusses challenges such as toxicity, targeting efficiency, and the need for scalable synthesis, alongside the limitations of computational modeling in capturing complex biological environments. Emphasizing the importance of ongoing research and interdisciplinary collaboration, this review underscores the potential of integrating computational insights with AuNP technology to enhance the precision, safety, and effectiveness of therapeutic and diagnostic approaches.

## 1 Introduction

A large number of biological industry sectors employ metal nanoparticles because of their modest size-to-volume ratio and high thermal stability. Due to their minimal toxic effects, clarity of detection, ease of manufacturing, stabilization, and functionalization, gold nanoparticles are an obvious option for biological uses (1). Among various nanomaterials, nanoparticles have the opportunity to act as nanocarriers simply due to their improved solubility, prolonged release, targeting capabilities, and ability to give medication dosage reductions (2). Gold nanoparticles are promising delivery systems for proteins, genetic elements, medicines, and small molecules (3). The most fascinating nanomaterial has been thought to be gold nanoparticles due to their special optical, electrical, sensing, and biological characteristics (4). Many vehicles have been developed in the last ten years using various nanomaterials including polymers, dendrimers, liposomes, nanotubes, and nanorods. Recently, gold nanoparticles have become a popular option for delivering multiple payloads to their targets (5). The efficacy of the therapeutic agents' release is essential; these agents might be tiny medication molecules or huge macromolecules like proteins, DNA, or RNA for effective therapy (6).

### 1.1 Gold nanoparticles (AuNPs) and their types

**Figure 1:**
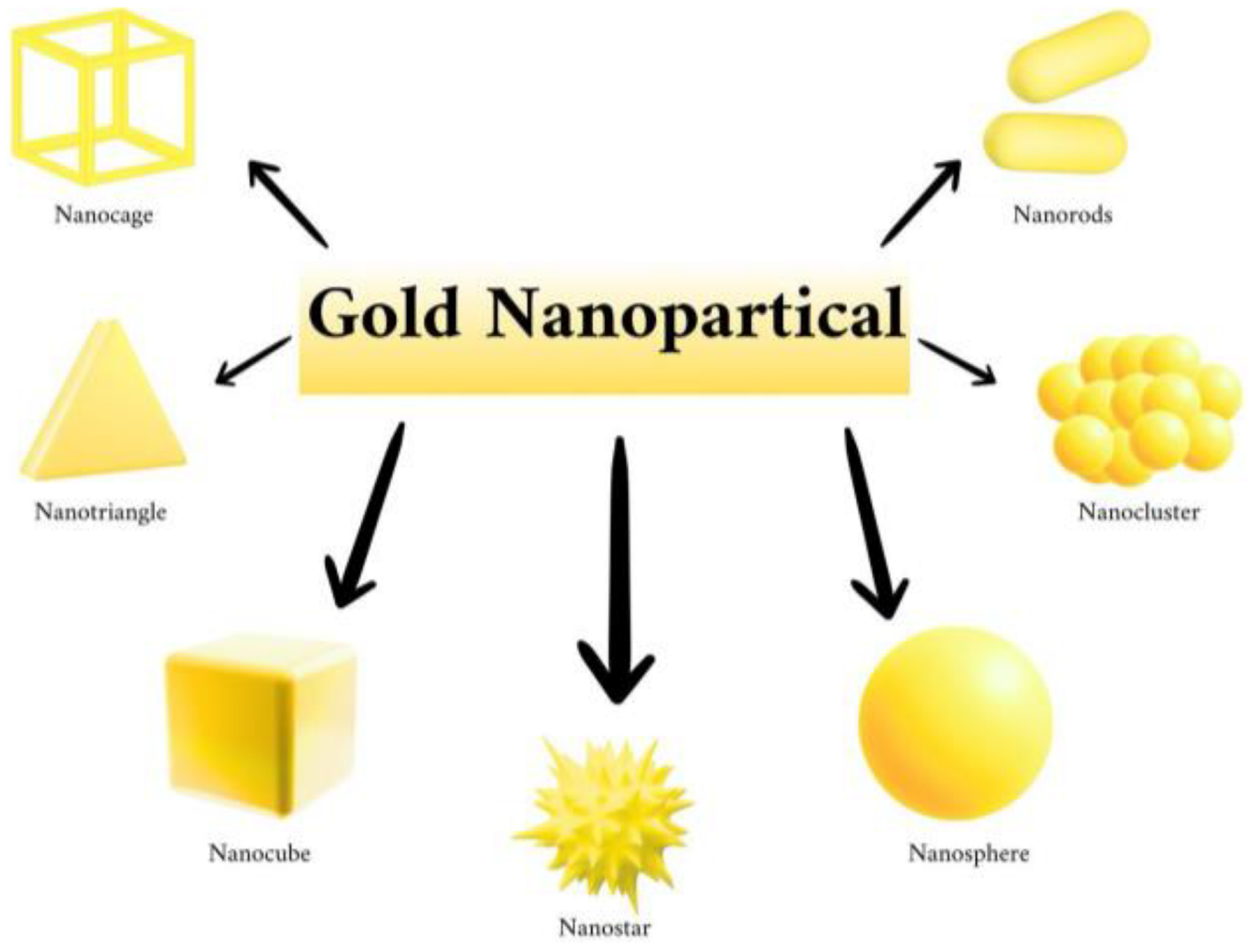
Types of gold nanoparticles (AuNPs), created with bioRender.com.

Gold nanoparticles (AuNPs) may be separated into three types according to their dimension: (Figure 1).

One-dimensional AuNPs: Nanorods, nanowires, nanotubes, and nanobelts.

Two-dimensional AuNPs: Gold nanoplates in the shapes of stars, pentagons, squares, rectangles, hexagons, truncated triangles, and dimpled nanoplates.

Three-dimensional AuNPs: Branching AuNPs like nanopods, nanostars, and gold nanodendrites, as well as gold nanotadpoles and gold nanodumbbells (AuNDs).

Plasmon absorption occurs in the visible and near-infrared (NIR) regions, which are especially influenced by the shape of the AuNP due to the unique characteristics of these irregularly shaped hollow and nano-shell AuNP structures. This characteristic has led to the development of medical applications including treatment and diagnostics. Because they are so simple to make, spherical or quasi-spherical gold nanoparticles have drawn the most interest. These spherical forms are widely studied due to their ease of synthesis and the potential for functionalization, which makes them suitable for various biomedical applications. The unique plasmonic properties of AuNPs, influenced by their shape and size, enhance their utility in targeted therapies and diagnostic imaging, providing significant advantages in the field of nanomedicine (7).

### 1.2 Drug development and computational methods

**Figure 2:**
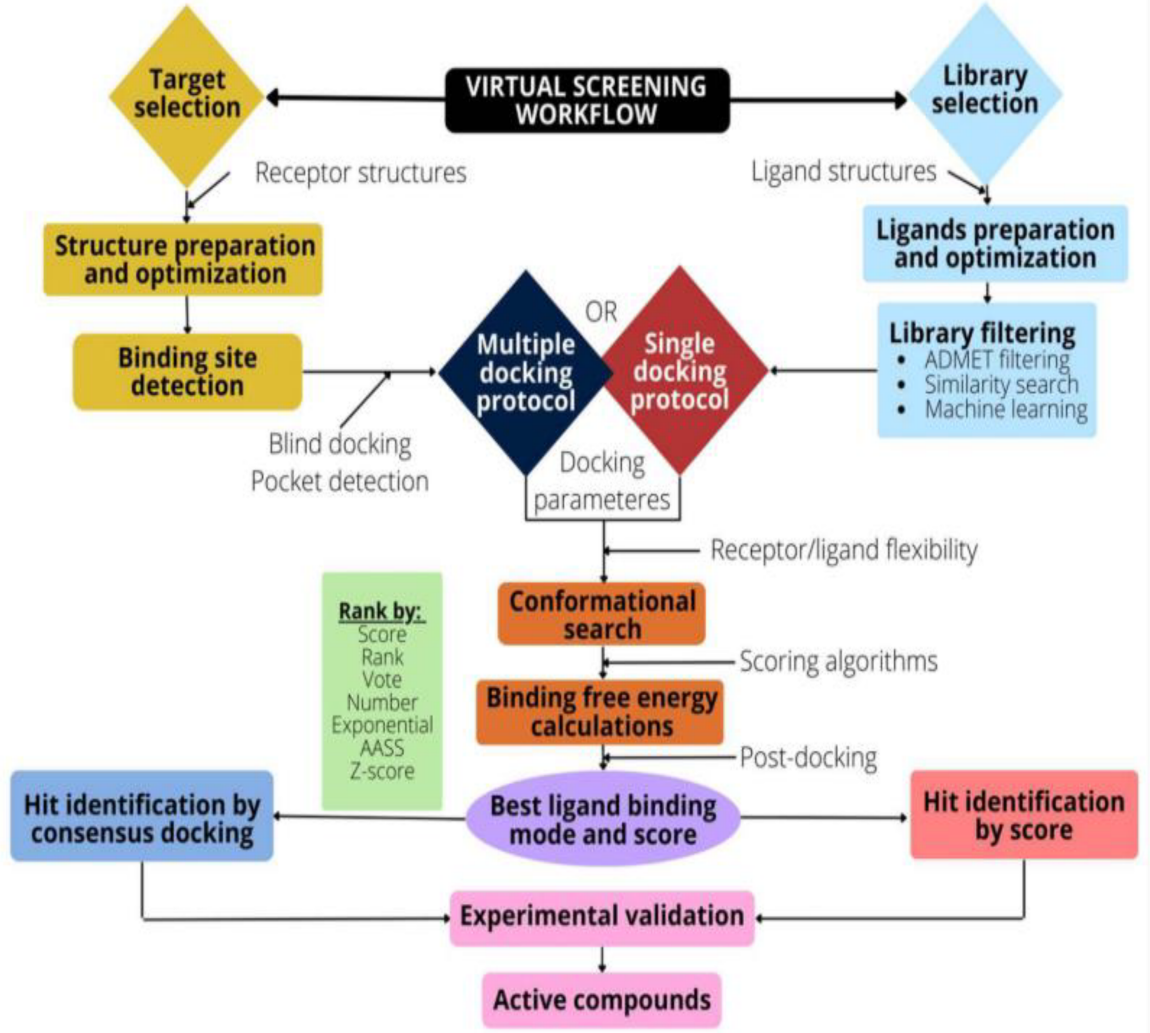
Flow diagram representing virtual screening workflow. The target selection, structure preparation and optimization of the binding target (receptor), the binding pocket identification, and preparation of the docking parameters. Library selection involves optimization and preparation of desired ligand structure. An ADMET screening or similarity search can reduce the ligand space. The poses, and scores are determined by docking algorithms. Further, the post-docking analysis viz., consensus docking, ameliorates the performance of the VS technique. Permission under open access Creative Common CC BY license (9). The target selection and optimization is main step where target of interest are instantly picked up from protein databank reliable for VS and to accomplish productive docking. The prediction of binding site, selection of libraries, availability of docking protocol, rescoring and post-docking analysis are another important steps involved in VS.

The pharmaceutical industry heavily relies on the drug development process. Advancements in computational resources and small molecule databases have greatly improved the identification of lead compounds. The increasing number of drug targets has led to widespread use of computational methods to expedite the drug discovery process. Consequently, there's been a surge in the application of computer-assisted drug design and chemical bioinformatics techniques, including high-throughput docking, homology search, and pharmacophore search in databases for virtual screening (VS) technology (8). The process of virtual screening is essential in computer-aided drug design methods. It provides a cost-efficient approach to discovering potential lead compounds and has demonstrated success in numerous instances (8). High throughput screening (HTS) is an experimental screening of vast libraries of molecules to disclose the hit-target for desired biological activity, play pivotal role in the drug development process. For instance, an automated patch-clamp or microfluorography are HTS technique often used to accelerate the screening of lead molecule libraries on ion channels (9) (Figure 2). The recently emerged technique, automated patch-clamp is used to ameliorate sealing capacity of the cell surface and the perfusion system. Moreover, fluorescent dyes are used to monitor change in ion concentration in the cytoplasm or membrane voltage due to ion channel activity through microfluorography. However, HTS is not up to the expectation in generating lead compounds speedily as required probably due to due to slow screening process, chances of aggregation, and target system (10, 11). The limitation of HTS technique led to development of rational-based approaches to filter tens of millions of compounds.

Homology modelling predicts the protein structure by aligning their sequence to a homologous protein that creates a template for model the construction. The homology prediction divided into three parts: template identification; sequence-template alignment; and model construction. The protein sequence is initially identified from data base Universal Protein Resource (UniProt) or experiment accompanied by identifying modelling template with high sequence similarity against Protein Data Bank.

VS technology is an in silico method implemented in the scientific drug discovery (10, 12). Adopting VS in computational technique, a large number of molecular structure databases assessed mechanically. Implementing VS technique enabling to identify the lead molecules more prone to get binding affinity to the molecular target such as protein, ligands, or enzyme (13–15). It works as filter to screen out the final lead drug compound in very less number within the vast number of molecules.

The lead compound having some properties alike to drug with known mechanism of action can screened to process. Then, the ligand molecules are analyzed based on the pharmacophore group, which is free of any kind of toxicity. In the adjacent step, the best featured ligand candidates are key out demonstrating the chemical structure and composition and, any modification to enhance the ligands properties. The ligand characteristic such as; the pharmacokinetic properties; absorption, distribution, metabolism, excretion, and toxicity (ADMET)). The ligand composition and structure can further be re-assessed for finalizing ligand candidate for biological assay (16).

Tran-Nguyen and associates, as of late experimented on unbiased investigation of 4- scoring functions to re-score and docking poses of collecting data for a high-confidence screening addressing many drug targets. They observed that rescoring relied on simple knowledge-based scoring functions, viz., fingerprints interaction measurement, seems to surpass the modern machine learning technique, signifying the use of rescoring methods for potent binder selection (17).

Computational simulation tools, including computational fluid dynamics (CFD), dissipative particle dynamics (DPD), coarse-grained (CG) molecular dynamics (MD) modeling, quantum mechanical methods, atomistic molecular dynamics, quantitative structure-activity relationships (QSAR), discrete element modeling, pharmacokinetic/pharmacodynamic modeling (PK/PD), and physiologically based pharmacokinetic (PBPK) modeling, are crucial for comprehending and predicting the self-assembly behavior of drugs, molecular interactions, and biological activities of nanoparticles during formulation design. These computational models of molecules help in understanding the intricate phenomena associated with the development of nanoparticle formulation (18, 19). In the computational study of the interaction between charged nanoparticles (NPs) and model lipid membranes, atomistic models are considered the most reliable choice (18, 20). This is primarily due to their ability to provide a more accurate treatment of electrostatic interactions, making them preferable for this type of analysis (18, 20).

### 1.3 Molecular dynamic simulations and nanomedicine

Molecular dynamics simulation is a sophisticated computational method used to examine the formation and structural alterations of nanoparticles under various conditions. This technique aids in modifying nanoparticles by integrating polymers and chemical functional groups to enhance their pharmacokinetic properties, minimize toxicity, and improve targeted drug delivery (19).

The current advances in nanotechnology field and their applications in manufacturing nanomedicines for targeted or site-specific drug delivery brought a new hope in nanoparticle for drug discovery and pharmaceutical uses (21). At present, numerous types of nanoparticles of lipid, polymeric, metallic, organic, inorganic, carbon nanotubes etc. with desired physical characteristics are being scientifically enquired for pharmaceutical uses (22, 23). As we know, the typical nanoparticle size varies between 1 and 100 nm or more. The molecular simulation study is well applicable to nanoscale particle for investigating its molecular structure and various other properties including drug load, entrapment efficacy, drug release mechanism, interaction with biological cell membrane and drug penetration capacity through cell membrane (23–26).

The molecular dynamic simulation basically evolved from Newton's second law (27). When the particle in motion, the total energy is an additive of the kinetic and potential energy of individual particle in a system. Considering the atomic system in motion with definite trajectory as Newtons second law. Briefly, MD simulation ascertains the atom's potential energy from the coordinates axis of each atoms and then predicts the position of the atoms in the succeeding moment, which is analyzed through statistical methods (28). Force field is an important in MD simulation process as it defines the sets of function of internal coordinates of particle in a system. The commonly used force field in pharmaceuticals or biomedicines are All-atom force fields (29) (first category) viz., chemistry at harvard macromolecular mechanics (CHARMM), assisted model build-ingand energy refinement (AMBER). Second category is United-atom force fields such as The GROMOS force field (30). Coarse-grained force fields are the third category (31).

Samaneh and co-author experimented et al. (32). on hexadic m-phenylene ethynylene (m-PE) macroing tubular assemble structure, to inquire the capability of 10- macrocyclic self-assembly nanotubes in organic and aqueous solvents. MD simulation implemented here to observe the dual transport of anticancer agent Curcumin and Doxorubicin on hexamer nanoscale carrier self-assembly system. The MD simulation specific process followed were; generate the initial model with the CHARMM force field, and applying steepest descent technique for energy minimization, and then, conserve temperature and pressure under the isothermal and isobaric (NPT) ensemble. The findings headed towards significantly high Dox loading on the self-assembled carrier system due to co-existence of the Curcumin drug. The dual loading of Dox and Cur in macrocyclic self-assembly nanotubes greatly reduced cytotoxicity, and which could be used develop a potential drug delivery system than CNTs nanostructures.

The pharmacokinetic- pharmacodynamic (PK-PD) model is essential in new drug development and has been effectively applied to study traditional dosage forms, such as injectables and infusions, in ADME and toxicity research over the last forty years. To address the challenges in clinically translating nanodrugs, PK-PD models tailored to nanomedicine have been created (33).

Nanomedicine facilitate personalized targeting by coating the surface of drug-loaded nanoparticles with specific ligands, such as antibodies, membrane-bound receptor ligands, and other cellular markers. This allows the nanoparticles to selectively bind to target cells when they are near the target tissue. Integrating computational modeling into the nanoparticle design phase is crucial for enhancing the success rate of targeted therapies. For instance, a computational model using a Metropolis Monte Carlo algorithm calculated the optimal antibody surface coverage for nanoparticles to bind specifically to vascular endothelium. This model calculated the binding free energy of nanoparticles by starting from a randomly selected state of the nanoparticle and ligand system and making random changes to the defined variables, thus generating a probability distribution of the system's configurations (34).

## 2 Gold nanoparticles (AuNPs) and use of computational methods

AuNPs are utilized as drug carriers because of their inert nature, non-toxic properties, and straightforward synthesis process (35). The application of computational methods for designing optimal gold nanoparticles (AuNPs) offers a rapid and cost-effective approach to supplement experimental work and offer valuable directives. Gold nanoparticles (AuNPs) have emerged as a promising platform for cancer diagnostics and therapeutics due to their unique physicochemical properties (35). These nanoparticles can be easily functionalized with various biomolecules, including drugs, targeting ligands, and genes, making them excellent candidates for drug delivery applications. Specifically, the presence of a negative charge on the surface of gold nanoparticles allows for easy modification and the incorporation of a wide range of therapeutic agents (35). Computational modeling techniques have been increasingly used to study the interactions between AuNPs and pharmaceutical molecules, providing valuable insights into the behavior and properties of these complex systems (35).

Colangelo E, et al., developed computational model of peptide-capped Au nanoparticles using experimentally characterized Cys-Ala-Leu-Asn-Asn (CALNN)- and Cys-Phe-Gly-Ala-Ile-Leu-Ser-Ser (CFGAILSS)-capped Au NPs. The monolayers structure of CALNN and CFGAILSS was determined with molecular dynamics simulations and structural biology approach. The finding revealed that monolayer structure dependency on the peptide capping density as well as on the nanoparticle size established by the model. Moreover, the computational findings showed new properties of peptide-capped monolayers could help in developing predictive tool in designing bio-nanomaterials (36).

Vorobyova et al. (37) synthesize a gold nanoparticles (AuNPs) with the plum peel extract using deep eutectic solvent (DES) comprising Betaine-Urea and Choline chloride-Urea. The quantum chemical technique was used to investigate mechanism of preparation of DESs and molecular interactions among components. The reducing ability of extract was 489–580 mg ASE/g extract recorded vis-à-vis to 336 mg ASE/g extract using conventional technique. The AuNPs synthesised herein showed significant antioxidant capacity and enhanced antimicrobial activity against Escherichia coli and Bacillus subtilis (37).

Further, a study exemplified how computational approach is important in designing a AuNP as a drug carrier. A computational approach is rapid, economic and provides important insight over an exhaustive or expensive and time-consuming experimental process. Herein, authors performed atomic MD simulation to analyse how the particle size, hydrophobicity, and drug concentration impacted the structure of functional AuNPs in the aqueous medium. The two groups of the functionalized nano-system simulated using a zwitter ion ligand as a background, and drug carrying ligand (Quinolinol or Panobinostat). Results showed that hydrophobicity causes conformation alteration in the coating layer of Quinolinol. The surface of hydrophobic drug adsorbed less solvent and coating thickness thus reduced resulting lesser particle size. Oppositely, the water soluble hydrophilic drug, Panobinostat, the ligand in excess has a superior impact on the final coating structure and drug access is higher than hydrophobic systems. It entails generally the greater biological activity for hydrophilic systems. The results interpreted that the physico-chemical properties of drugs and ligands play significant role in while developing AuNP-based nanosystems of hydrophobic drugs (38).

Considering immunotherapy as promising therapeutic approach in cancer and to boost immune system in response to therapy, Ali et al., aimed to block programmed cell death protein 1/Programmed death-ligand 1 (PD1/PDL1), which stimulates T cells. Herein, computational tools were used to find high-affinity drugs against PDL1 and then conjugate nanoparticles with them. The cytotoxicity of the drug conjugated nanoparticle drugs was investigated. Ten thousand ZINC database and FDA approved drugs were filtered computationally. The physicochemical features and drug toxicity were investigated with SwissADME and ProTox-II. Silver and gold nanoparticles were synthesized using extracts and characterized by UV–Vis spectroscopy, XRD, and FTIR. The filtered drugs were Irinotecan, Imatinib, and Methotrexate. Docking experiments showed that Irinotecan had the prominent binding affinity for PDL1 when conjugated with silver and gold nanoparticles. The MD simulation also revealed the most stable Irinotecan-PDL1 complex. The cytotoxicity also showed that Irinotecan-conjugated silver and gold nanoparticles had higher toxic effect on the A549 cancer cell line than Imatinib conjugated silver and gold nanoparticles (39).

Furthermore, computational modeling can help in the rational design of gold nanoparticle-based drug delivery systems by allowing for the optimization of parameters such as nanoparticle size, shape, and surface functionalization. For example, studies have shown that the size of gold nanoparticles plays a significant role in their biodistribution and tumor targeting capabilities (35). Computational simulations can be used to predict the optimal nanoparticle size for enhanced tumor accumulation and drug delivery (35).

One study used molecular dynamics simulations to investigate the interactions between AuNPs and doxorubicin, a commonly used chemotherapy drug. It was found that the size and shape of the AuNP significantly affected the binding affinity and release of the drug, highlighting the importance of considering nanoparticle characteristics in drug delivery applications (35). Another study utilized quantum mechanics calculations to explore the interactions between AuNPs and various anti-inflammatory drugs. The researchers found that the presence of AuNPs enhanced the binding affinity of the drugs, potentially leading to improved drug delivery and therapeutic efficacy (40). In a more recent study, molecular docking simulations were employed to investigate the interactions between AuNPs and small-molecule inhibitors of HIV protease. The study revealed key binding sites and interactions between the AuNPs and the inhibitors, providing valuable insights for the design of novel antiviral agents (41). Overall, computational methods offer a powerful tool for studying the interactions between AuNPs and pharmaceutical molecules. By providing a detailed understanding of these complexes at the molecular level, computational modeling can guide the design and optimization of AuNP-based drug delivery systems.

### 2.1 Mechanisms of cellular uptake and computational insights

The cellular uptake of gold nanoparticles (AuNPs) is influenced by various factors, including size, morphology, surface chemistry, and aggregation (42). The size of AuNPs determines their uptake pathway, with large nanoparticles entering cells via phagocytosis and small nanoparticles directly translocating through the cell membrane (42). The pathway of phagocytosis is size-dependent, with optimal and minimum encapsulation diameters essential for successful phagocytosis, although the exact minimum size remains unclear (42). Non-spherical gold nanostructures, such as nanorods, nanoplates, nanocages, and nano-hexapods, exhibit unique cellular uptake properties due to their asymmetric shapes, making them effective in photothermal therapies (42). The surface chemistry of AuNPs also plays a critical role in their internalization pathways, with clathrin-mediated cationic gold nanoparticles entering the cell via phagocytosis and spherical gold nanoparticles utilizing dynamin-dependent pathways (42). The targets of several drug molecules are localized within subcellular spaces. The nanoparticle-cell interaction for potential cell uptake via plasma membrane and the developing nanoparticle with in-build feature for precise cellular and subcellular delivery remains challenging. The post internalization feature and intracellular localization determined by cellular internalization (43). One study reported that nanoparticle shape is important to study. Shape can regulate nanoparticles uptake into RAW264.7 cells. The triangle shaped nanoparticles were most dominantly undergo cellular uptake, and thus it furnishes insight guidance for designing carrier system for drug delivery (44). Additionally, the aggregation of gold nanoparticles affects their cellular uptake, with non-aggregated gold nanoparticles being internalized more efficiently than aggregated ones, likely because they behave as spherical particles and do not use endocytic pathways (42).

Computational methods provide important perceptions and significant observations into the mechanisms of endocytosis and intracellular transport of AuNPs. Coarse-grained molecular dynamics (CGMD) simulations have been utilized for studying interactions between mammalian plasma membranes and gold nanostructures, revealing the complexities of lipid bilayers on nanoparticle uptake pathways (45). These simulations have shown that the state of aggregation significantly decreases the uptake of gold nanoparticles (45). The morphology of gold nanostructures affects transport rates, with hexapod nanoparticles showing higher permeability and being more suitable for applications such as photothermal conversion. Electrostatic interactions between negatively charged membranes and positively charged gold nanoparticles drive the cellular phagocytosis process, especially for smaller nanoparticles. However, excessive positive charge can hinder uptake due to strong interactions that prevent vesicle formation. Neutron reflectometry (NR) and atomistic and coarse-grained molecular dynamics (MD) have demonstrated interactions between cationic gold nanoparticles and model lipid membranes composed of zwitterionic di-stearoyl-phosphatidylcholine (DSPC) and anionic di-stearoyl-phosphatidylglycerol (DSPG) (42). Understanding these interactions at the molecular level through computational modeling aids in the design and optimization of AuNP-based drug delivery systems (45). Alla P. Toropova, et al., implied computational models in gold nanoparticles uptake by A549 cells under distinguished condition. The computational tool used were simplified molecular input-line entry system (SMILES) to show the molecular structure with symbols, and quasi-SMILES to determine the physicochemical and biochemical conditions both. Finally, they enable to developed statistically defined models of gold nanoparticles for the potential uptake in A549 cell (46).

Sung W, et al., evaluated radio enhancement of gold nanoparticles (GNPs) intending improved radiotherapy. They applied computational approach towards radiation response, and predicted the survival rate in breast cancer when incubated with gold nanoparticles. Further, the nanoparticle uptake was estimated by implementing coupled plasma-mass spectroscopy, and the three-dimensional (3D) intracellular distribution of nanoparticles was prevailed by optical diffraction tomography. Here the computational model used the 3D live cell imaging and Monte Carlo method to determine the dose distributions in the cell. The measured cell survival rate was found comparable with the computational model (47).

### 2.2 Influence of lipid composition on gold nanoparticle interaction

Negatively charged lipids, such as phosphatidylserine (PS) and phosphatidylglycerol (PG), are present in cell membranes. PS is typically found in the inner leaflet of the plasma membrane, contributing to the negative charge on the inner surface (48). Phosphatidylinositol (PI) and its phosphorylated derivatives (PIPs) also bear negative charges and have significant roles in cell signaling. Using neutron reflectometry (NR) and atomistic and coarse-grained molecular dynamics (MD), researchers have shown the interaction between cationic gold nanoparticles (AuNPs) and model lipid membranes composed of a mixture of zwitterionic di-stearoyl-phosphatidylcholine (DSPC) and anionic di-stearoyl-phosphatidylglycerol (DSPG). When exposed to a water-based solution containing positively charged Me3N + AuNPs (0.01 mg ml^−1^) at 25 degrees Celsius, no noticeable alterations in the reflectivity profile were observed (49).

However, increasing the temperature to 53°C, which is still below the gel-fluid phase transition temperature, caused the floating bilayer to become destabilized in the presence of cationic AuNPs. This destabilization highlights the sensitivity of lipid bilayers to temperature changes and nanoparticle interactions. The presence of cationic AuNPs caused noticeable differences in the behavior of the negatively charged DSPC/DSPG (3:1) bilayer compared to pure DSPC bilayers. When cationic AuNPs were added to the solution and interacted with the DSPC/DSPG bilayer, the reflectivity profile showed that a layer of AuNPs adhered to the floating lipid bilayer due to attractive interactions between the positive terminal groups of the AuNPs and the negatively charged lipid head groups (45). In contrast, within a bilayer consisting solely of DSPC, positively charged AuNPs could effectively integrate into the membrane with minimal disruption. When in a bilayer composed of DSPC and DSPG, the behavior of the positively charged AuNPs changed, as they tended to remain on the surface or have loose associations with the membrane despite being attracted to the negative charge of the DSPG lipids (49).

Research has utilized molecular dynamics simulations to model the interaction between chitosan and the lipid bilayer, which is similar to the bacterial membrane (48). The main aspects of the modeling include:

 Simulation parameters: GROMACS 4.5.3 was used considering Lennard-Jones potential with van der Waals forces and Ewald particle mesh method with electrostatic interactions, the simulation was performed at 1 atm and 350 K while ensuring that the lipid bilayers were in a liquid crystalline state (48).
 Lipid bilayer model: A symmetric bilayer composed of dipalmitoylphosphatidylserine (DPPS) and dipalmitoylphosphatidylcholine (DPPC) was used, where dipalmitoylphosphatidylcholine represents 20% of the molecular weight in the bacterial membrane model. This structure represents the charged bacterial cell membrane.
 Chitosan model: Two chitosan models were simulated representing different degrees of amine protonation, namely CT + 5 and CT + 1. The interaction between the lipid bilayer and chitosan was studied by placing chitosan models in simulation boxes containing water molecules, essential ions, and the lipid bilayer (48).
 Simulation results: The study provided predictions about the possible mechanisms by which the cesium-gold nanoparticles exert their antibacterial effects. The results indicated that the association and interaction with the lipid bilayer of chitosan is affected by the protonation state of chitosan, which affects the structural and mechanical properties of the membrane (48).

### 2.3 Reaxff molecular dynamics simulations

Reactive Force Field (ReaxFF) molecular dynamics simulations are a powerful computational approach used to model and understand the structure and dynamics of complex molecular systems. These simulations have been used to investigate the behavior of gold nanoparticles functionalized with chitosan and loaded with gentamicin, a potent broad-spectrum antibiotic (50). The interaction between chitosan and gentamicin with gold nanoparticles is mainly influenced by electrostatic interactions and hydrogen bonding. Chitosan molecules, which contain positively charged sites, bond with the negatively charged gold nanoparticles and gentamicin molecules, prompting the creation of a stable conjugate. This bonding ensures that gentamicin is effectively attached to the nanoparticles, enabling a controlled release mechanism (50). Experimental trials demonstrated that the antimicrobial effectiveness of gentamicin was maintained, with a slower release and gentler dosage in comparison to the free drug. This indicates that adjusting the chitosan/gentamicin weight ratio and deposition pattern could further improve the drug release profile (50). The nanoparticles' antimicrobial activity has been tested against both Gram-positive (*Staphylococcus aureus*) and Gram-negative (E*scherichia coli*) bacteria, showing effective inhibition. Tests for cytotoxicity using human epithelial cell lines demonstrate that the conjugated nanoparticles are safe for use at various concentrations (50).

## 3 Application of MD simulation and nanoparticle technologies in drug delivery

At present, in diverse array of biomedical fields, including tumor therapy, nanoscience has shown tremendous growth and evolving day-to-day. Nanoscience dealing with nanoscale particle working tiredly in various areas of which nanoparticles has wide range of application including drug delivery, therapeutic, diagnostic and imaging (51). Nanoparticles are a product of the technological adjustment in biomaterial leading to particles of different sizes, and shapes. Due to wider characteristics including stability, self-reassembly, easily adaptable and which can have altered easily for favorable or intended characteristics e.g., uniform size distribution, high surface area in comparison with conventional substances. The nanotechnology provided unique physico-chemical properties to the nanoparticles through altering their surface chemistry, and size and surface can easily have tuned for delivery of nanomedicine (52, 53). MD simulations are broadly executed on various drug delivery systems (DDSs) to image their features and the intermolecular interactions amongst the biological components such as drugs, therapeutics, RNA, DNA, bilayer (lipid-protein) membrane, polymers, peptides, nanoparticles, and solvents.

The computational technique providing information associated with structure change, formation, and physiochemical behavior of nanoparticles in different experimental conditions. The application of MD simulation in nano drug delivery systems resulted in exciting experimental outcomes and interpreted result data enormously help to predict the material of choice experimental conditions (19).

Choodet C, et al., developed an amphiphilic molecules of poly(lactic-co-glycolic acid)–poly(ethylene glycol) methyl ether (PLGA-MPEG) as a drug delivery carrier. In current study, MD simulations implemented to tailor polymeric surface of nanoparticles with enhanced drug delivery. The drug loading was customized using copolymers varying ratio of lactic acid to glycolic acid and suitable selection of MPEG chain length. The simulations were done with varying concentration of the drug and copolymers. Further, the strong interaction between gemcitabine drug and the glycolic acid-rich copolymer proposed that the drug might have a slow-release. At the similar way, it was observed that the copolymer's flexibility raised as the glycolic acid concentration in the copolymer increased, boosting nanoparticles characteristics for high drug encapsulation and release profile (54).

A Molecular Dynamics Investigation of Dendro[60]fullerene as a Nanocarrier for Molnupiravir” explores the potential of using fullerene-based nanocarriers for targeted drug delivery, particularly in treating viral infections like COVID-19. By employing molecular dynamics simulations, the researchers investigated the effectiveness of Dendro[60]fullerene, a water-soluble fullerene C60 derivative, in delivering the antiviral drug Molnupiravir (55).

In this system, nitrogen-nitrogen bonds were used to link Molnupiravir molecules to the nanocarrier, selected for their low dissociation energy, which allows for precise and controllable drug release. The study's findings demonstrated that the Dendro[60]fullerene-based nanocarrier maintained its high solubility in both water and n-octanol, showcasing promise for efficient delivery in biological environments. Notably, the addition of Molnupiravir did not significantly affect the nanocarrier's solubility, reinforcing its potential as a stable and effective vehicle for drug delivery (55).

These insights into fullerene-based systems highlight a growing trend of integrating nanotechnology with antiviral treatments, which aligns with current research interests in enhancing drug delivery mechanisms. This research supports the exploration of innovative nanocarrier systems, such as gold nanoparticles (AuNPs), in targeted therapies, emphasizing the importance of developing effective strategies to improve therapeutic efficacy against infections like COVID-19 (55).

## 4 Applications of gold nanoparticles in drug delivery

Gold nanoparticles have special physicochemical properties that make them potentially medicinal. Gold nano constructs are slowly but progressively making their way into clinical trials following two decades of preclinical advancement. While gold nano formulations were once believed to be “magic golden bullets” that could be used to treat a wide range of ailments, the current consensus has shifted toward a more practical approach, wherein specific conditions are being studied for treatment with gold nano formulations. The pharmacokinetics and biodistribution patterns of gold nanoparticles determine their medicinal uses (56). Because of their localized surface plasmon resonances—a collective oscillation of their conduction band electrons upon interaction with particular wavelengths of light—AuNP exhibits distinctive optoelectronic features (57). Through colloidal chemistry and engineering, these optoelectronic characteristics can be optimized. Nanocrystal size and shape (58). For instance, anisotropic gold nanoparticles exhibit high extinction coefficients in the near-infrared region, which allows for deeper tissue penetration of light (58–62). Notable examples of these nanoparticles include thin gold nanoshells, high-aspect-ratio gold nanorods, and gold nanostars (58–62).

### 4.1 Advanced computational techniques in drug delivery and drug release

Gold nanoparticles have become central to biomedical innovation, particularly in drug delivery and therapeutic applications. Advanced computational techniques play a crucial role in understanding and optimizing these applications. Notably, computational methods have made significant contributions in two key areas: modeling the mechanisms of Low-Intensity Pulsed Ultrasound (LIPUS)-induced drug release and developing frameworks that enhance pharmacokinetic modeling and toxicity assessments across diverse species (63, 64).

In this context, Low-Intensity Pulsed Ultrasound (LIPUS) has emerged as a promising tool for enhancing the release of chemotherapeutic drugs from gold nanoparticles. Several advanced computational models have been developed to explore this phenomenon. These models utilize Multiphysics techniques, incorporating acoustic, thermal, and mechanical fields to simulate the complex interactions between LIPUS and gold nanoparticles (63, 64).

The mechanisms by which LIPUS enables drug release are very diverse. The acoustic waves coming from LIPUS produce cavitation and acoustic streaming within the medium surrounding the nanoparticles (63, 64). The generation of microbubbles known as cavitation in response to ultrasound may result in locally changing pressure values and mechanical forces, giving rise to disruption on the surface of nanoparticles and an increased release of the drug. Acoustic streaming, or the steady flow induced by the ultrasound waves, may also help in dispensing the released drugs from the nanoparticle surfaces, increasing their bioavailability (63, 64). Another critical factor is thermal effects of ultrasound. This local heating may alter the permeability of the nanoparticle coating or even cause phase transitions in the encapsulated drugs, thereby enhancing drug release. Computational models coupling these acoustic, thermal, and fluid dynamics phenomena will help further improve the basic understanding of the drug release process under LIPUS stimulation. Only then would such an integrated approach in the optimization of the parameters of ultrasound application be possible, maximizing therapeutic efficacy and minimizing side effects (63, 64).

The pharmacokinetics and toxicity of gold nanoparticles are complex and vary significantly across different species. Pharmacokinetic modeling employs mathematical models to predict the absorption, distribution, metabolism, and excretion (ADME) of nanoparticles. In this study, advanced computational methods are utilized and integrated to simulate these processes across various species, ranging from rodents to humans (65). By integrating physiological parameters specific to each species—such as blood flow rates, organ sizes, and metabolic enzyme activities—these models enable precise predictions of the behavior of gold nanoparticles across species. This approach allows for the simulation of scenarios involving the intravenous injection of gold nanoparticles, tracking their journey through the bloodstream, accumulation in organs, and subsequent clearance. These simulations are invaluable for designing nanoparticles with optimal drug delivery characteristics, ensuring effective targeting of tissues while minimizing systemic exposure and potential toxicity. This advanced modeling, coupled with species-specific simulations, enhances our ability to predict and optimize the pharmacokinetics and safety of gold nanoparticles, making them more effective and safer for clinical use (65).

Cross-species comparisons are essential for translating nanoparticle-based therapies from animal models to human applications (65). By integrating physiological parameters such as blood flow rates, organ sizes, and metabolic enzyme activities, these computational models enable accurate predictions of nanoparticle behavior across different species. This includes simulating scenarios such as intravenous injection, nanoparticle movement through the bloodstream, accumulation in organs, and eventual clearance (65). Such simulations are crucial for designing nanoparticles with optimal drug delivery features, ensuring they effectively target tissues while minimizing systemic exposure and potential toxicity (65).

Allometric scaling further refines these models by adjusting for differences in body size and metabolic rates among species (65). This approach also considers the interactions of nanoparticles with biological systems, such as protein corona formation and immune responses, which vary significantly across species (65). As a result, these studies can simulate potential adverse reactions and predict human dosages from pre-clinical data, making them invaluable in the safe and effective translation of nanoparticle therapies from animal models to human clinical use (65).

### 4.2 Design principles and characterization of nanoparticle interactions for drug delivery

Traditionally, designing nanoparticles for transdermal drug delivery has involved a lot of trial and error. However, computer simulations can greatly improve this process, saving time and money. These simulations help researchers understand how nanoparticles with different surface chemistries and patterns move through the skin's lipid bilayer (66). By using computational methods, scientists can optimize the properties of nanoparticles for skin delivery. They do this by screening nanoparticles based on how easily they pass through the lipid bilayer, using a technique called constrained molecular dynamics (MD) simulations. This initial screening is then verified with additional, unconstrained simulations to ensure the nanoparticles are effective in delivering model proteins into the skin (66).

The inside of the lipid bilayer is not uniform, and MD simulations allow researchers to calculate the energy needed for nanoparticles to move through it, referred to as the free energy of permeation, ΔG(z), along the bilayer's normal direction, z. A model called the nonhomogeneous solubility diffusion model is used to compute this energy by assessing the potential of mean force in a symmetrical bilayer system (66). During these simulations, researchers calculate the force applied at different points along the bilayer and average it over time to determine the potential energy (66).

To confirm these findings, unconstrained simulations are run for each nanoparticle as it interacts with the skin's lipid bilayer, lasting for about 6 microseconds under specific conditions (NPT). Key measurements, such as the projected area on the XY plane, the overall order parameter, and the area compressibility of the bilayer-nanoparticle system, are then reported (66).

Building on the understanding gained from simulations of nanoparticle permeation through the skin's lipid bilayer, the process of protein co-delivery is further refined. The secondary structure of the protein is sourced from the Database for Secondary Structure of Protein (DSSP), and its detailed 3D structure is obtained from the Protein Data Bank (PDB ID: 1HCH) (66). This atomistic protein model is then simplified into a coarse-grained (CG) model using the Martinize tool, which allows for efficient simulation (66). Additionally, the permeation of nanoparticles through a double-layered skin lipid bilayer is simulated using VMD software, further enhancing the insights into transdermal drug delivery mechanisms. These simulations provide a comprehensive understanding of how nanoparticles can effectively deliver both drugs and proteins through the skin (66).

The interaction between gold nanoparticles and PAMAM dendrimers is explored through both experimental and computational methods (67). Molecular dynamics simulations reveal the atomic interactions involved in the adsorption of PAMAM dendrimers to gold surfaces, focusing on how the thermodynamics of this process depends on the dendrimer's terminal functional groups (67).

Experimental data combined with computational predictions highlight several key aspects. For instance, a 2D free-energy plot for PAMAM–NH3 + shows regions of high-affinity interactions with the gold surface, indicated by blue areas, while red regions represent less favorable interactions (67). The global free energy minimum, characterized by a distance (Z) of approximately 4.1 Å and a dz of around −0.95 Å, marks the strongest interaction. Similarly, the free-energy landscape for PAMAM–OH, based on the distance between the dendrimer and the gold surface, mirrors the profile of the oxidized PAMAM–CHO system. In this configuration, an extended geometry favors contact between the core, amido, and terminal groups with the gold, similar to the interactions seen in the PAMAM–OH and –NH3 + systems (67).

Further analysis through thermodynamic decomposition reveals significant differences in the adsorption thermodynamics of charged dendrimers like PAMAM–NH3 + compared to neutral ones like PAMAM–OH. Free-energy calculations suggest that while all dendrimers exhibit high affinity for the gold surface, PAMAM–NH3 + shows the lowest affinity due to a strong enthalpic component that is offset by an unfavorable entropic component. Specifically, the enthalpic contribution for PAMAM–NH3 + is about 50 kcal/mol lower than that of other dendrimers, which may correspond to better stabilization of gold nanoparticles by this dendrimer (67). This enthalpic component is balanced by entropy changes driven by the reorganization of water molecules and ions, influenced by the charged nature of the dendrimer (67).

### 4.3 Case studies in drug delivery

#### 4.3.1 Example 1: AuNPs in targeted delivery of chemotherapeutics

Investigates the potential of gold nanoparticles (AuNPs) as effective drug delivery vehicles for doxorubicin, a widely used anticancer drug. Utilizing molecular dynamics simulations, the research examines how the functionalization of AuNPs with drug-binding peptides can enhance the targeted delivery of doxorubicin to cancer cells. The findings reveal that the conjugation of doxorubicin to the gold nanoparticles significantly improves its stability and delivery efficiency, allowing for better interaction with cancer cell membranes. However, the study also highlights challenges related to drug loading capacity, as increased drug amounts may lead to competition for binding sites on the nanoparticles, potentially resulting in the dissociation of drug molecules. Overall, this research underscores the promise of gold nanoparticles in improving the precision and effectiveness of cancer therapies by enhancing the delivery mechanisms of conventional anticancer drugs (68).

#### 4.3.2 Example 2: AuNPs for gene delivery in gene therapy

In a recent study, researchers developed polyethyleneimine-modified gold nanoparticles (AuNP-PEI) as effective carriers for gene therapy targeting triple-negative breast cancer (TNBC). The AuNPs were conjugated with siRNA targeting the eukaryotic elongation factor 2 kinase (eEF-2K), which is implicated in oncogenic pathways. The study demonstrated that this approach not only facilitated efficient cellular uptake of the siRNA but also resulted in significant gene silencing, leading to a marked reduction in tumor growth in both in vitro and in vivo models. These findings underscore the potential of gold nanoparticles as versatile platforms for enhancing gene therapy efficacy in cancer treatment (69).

## 5 Applications of gold nanoparticles in cancer therapy

Despite the significant progress made in medicine in recent decades, cancer remains a significant global health challenge, ranking as the second leading cause of death worldwide and resulting in nearly 10 million deaths in 2018 (WHO 2024). Given the substantial side effects associated with standard chemotherapy, the primary hurdles in cancer treatment revolve around achieving selectivity and precision in targeting cancer cells (70, 71).

**Figure 3:**
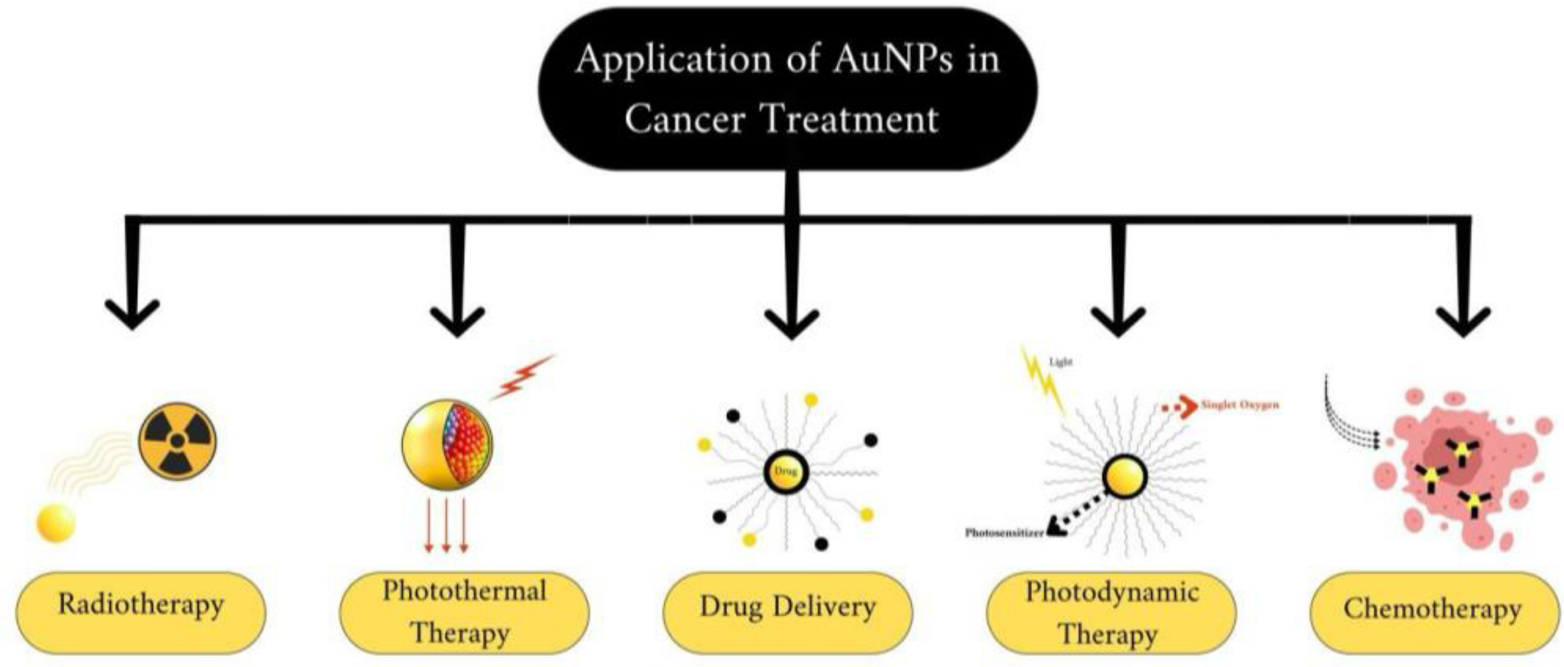
Application of gold nanoparticles (AuNPs) on cancer treatment, created with bioRender.com.

To address this challenge, drugs could be specifically targeted to cancer cells using vectors, thus mitigating the issue of side effects. Due to their distinctive optical and surface modification characteristics, AuNPs hold significant promise in various cancer treatments, including photothermal therapy (PPT), radiotherapy, photodynamic therapy (PDT), and drug delivery (70) Figure 3.

**Figure 4:**
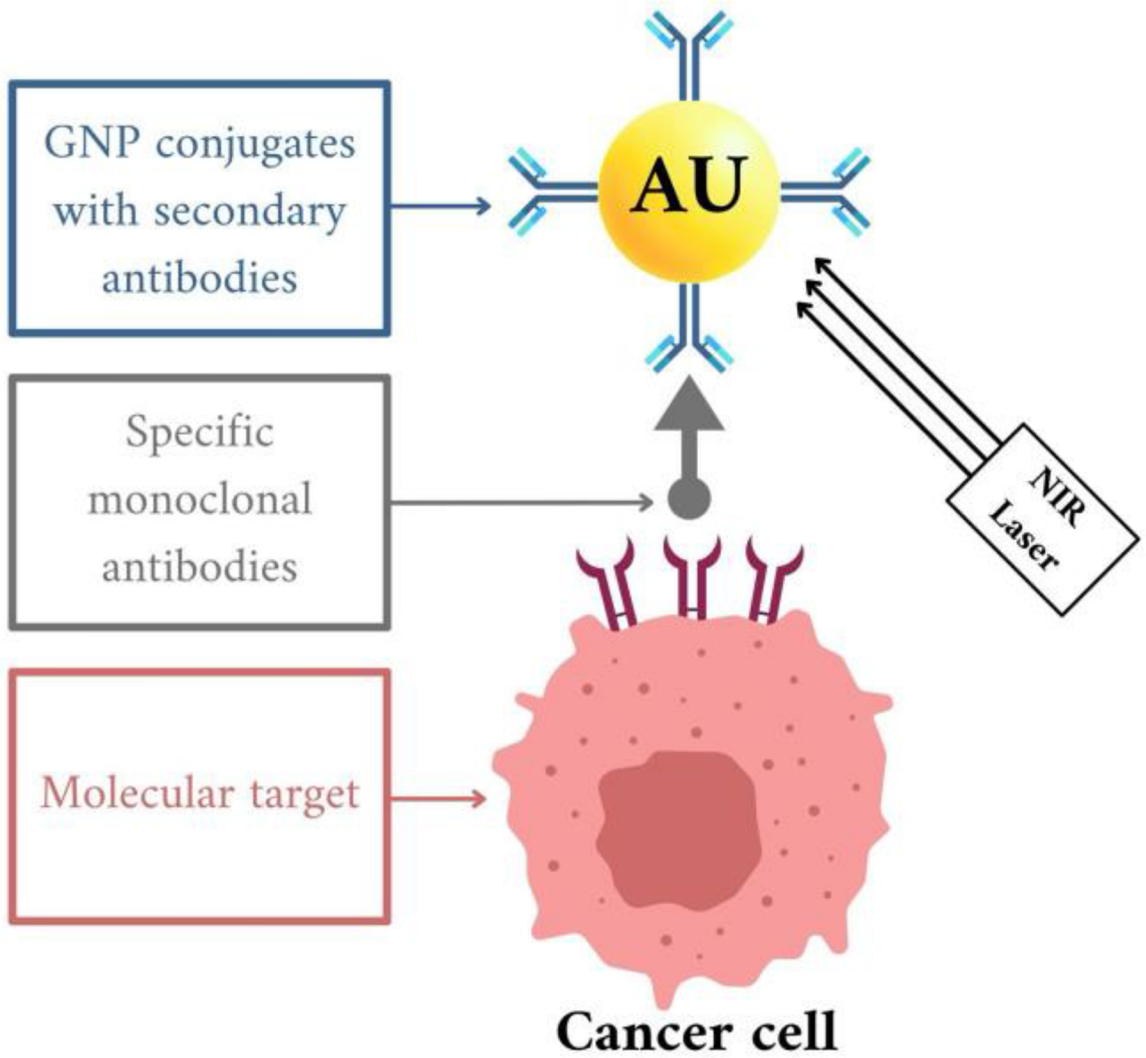
*In vitro* gold nanoparticle (AuNP) therapy for cancer cells, created with bioRender.com.

Photothermal therapy (PTT) represents a cancer treatment modality wherein nanoparticles are implanted into the tumor and produce heat upon exposure to externally applied laser light. Studies indicate that PTT exhibits considerable efficacy in treating cancer (70). Photothermal therapy (PTT) operates by converting light energy, typically in the near-infrared (NIR) region, into heat, thereby inducing cellular necrosis or apoptosis and leading to tumor ablation (67, 68). This targeted cancer therapy approach utilizes gold nanoparticles (AuNPs) conjugated with secondary antibodies, guided by specific monoclonal antibodies to the molecular targets on cancer cells. Once the AuNPs are bound to the cancer cells, a near-infrared (NIR) laser is applied to activate the nanoparticles, leading to the targeted destruction of the cancer cells. This method leverages the precision of monoclonal antibodies and the unique properties of AuNPs, enhancing treatment effectiveness while minimizing damage to healthy tissues (72, 73) Figure 4.

Another promising treatment is photodynamic therapy (PDT), a cancer treatment method that stands to benefit significantly from coupling with gold nanoparticles (AuNPs). PDT involves the use of light, photosensitizers, and tissue oxygen (74).

AuNPs can also be used for drug therapy. Conjugates of gold nanoparticles (AuNPs) with drug molecules are significant in treating intracellular diseases as they have the potential to enhance drug efficacy (4). Multiple studies have explored the utilization of gold nanoparticles (AuNPs) as carriers for tumor necrosis factor-alpha (TNF-α), a potent anticancer cytokine, and Methotrexate (MTX), a chemotherapy agent. These studies have shown that AuNP-based delivery systems can enhance tumor damage while reducing systemic toxicity (75).

AuNPs can have a significant impact on the effectiveness of chemotherapy drugs. By using AuNPs to deliver drugs to target sites in the body, the nonspecific side effects of chemotherapy can be reduced while still allowing for higher doses of drugs to be administered (76). The success of this approach is due to the high drug loading capacity of AuNPs and their low cytotoxicity, making them a promising tool in the fight against cancer (76). AuNPs are predominantly utilized in the field of oncology for cancer diagnostics and treatment purposes, and they take this process to another level by improving how drugs work and how gene therapy is delivered in the body (76). The lymph nodes are major sites of cancer metastasis; therefore, it is imperative to study the mechanisms of delivering molecules to the lymph nodes. To deliver the drug to the lymph node, it is necessary to overcome physiological barriers such as (1) avoid clearance from the bloodstream by the mononuclear phagocyte system (MPS), (2) extravasate out of fenestrated blood vessels, and (3) traverse the extracellular matrix of the interstitium (4). It has been discovered that the size of nanoparticles plays a significant role in their clearance from the body. Particles larger than 100 nm tend to have a shorter half-life in the bloodstream, and their ability to move through the extracellular matrix (ECM) diminishes as their size increases (76). Moreover, the shape of nanoparticles is crucial in determining their adhesion to vessel walls. Research indicates that nanoparticles with higher aspect ratios, such as ellipsoid or rod-shaped particles, have better margination to vessel walls compared to spherical nanoparticles (76). This understanding has led to the conclusion that larger and more complex-shaped gold nanoparticles (AuNPs) may offer greater anticancer potential due to their enhanced interaction with tumors, but this comes at the cost of reduced cellular uptake (76).

In the context of colorectal cancer treatment, recent studies suggest that combining AuNPs with the chemotherapy drug doxorubicin and an anti-PD-L1 antibody—an immune checkpoint inhibitor that blocks the PD-L1 protein to help the immune system attack cancer cells—can lead to more effective treatment outcomes. This combination leverages the unique properties of AuNPs to improve drug delivery and boost the immune response, providing a promising approach for cancer therapy (76).

Gold nanoparticles have the potential to be utilized in imaging and radiation-based therapies. They are able to interact with electromagnetic radiation, then create an image, which can be helpful in identifying tumors at a very early stage (76). The surface plasmon resonance (SPR) The unique properties of Gold Nanoparticles enable their utilization in various imaging modalities, particularly in near-infrared (NIR)-resonant imaging. These modalities include magnetic resonance imaging (MRI), photoacoustic imaging (PAI), positron emission tomography (PET), fluorescence imaging, and x-ray scattering imaging (x-ray CT) (76).

## 6 Recent advancements in gold nanoparticles

Researchers have investigated the synthesis and antimicrobial activity of antimicrobial peptide-conjugated gold nanoparticles (AMP-AuNPs). These AMP-AuNPs are highly effective against a broad range of pathogens, including bacteria and fungi, due to the antimicrobial properties of the peptides (AMPs) they carry. Their broad-spectrum efficacy, combined with a lower likelihood of resistance development, makes them a promising solution in the fight against various microbial infections (77). AMP-AuNPs are considered promising alternatives to traditional antibiotics. However, despite their benefits, antimicrobial peptides (AMPs) face challenges such as limited permeability and stability (77). To address these issues, researchers have demonstrated that using gold nanoparticles (AuNPs) as carriers for AMPs can effectively overcome these limitations. Conjugating AMPs with AuNPs not only enhances their stability but also improves their antimicrobial activity and target specificity. Various methods for functionalizing AuNPs with AMPs were explored, including covalent attachment, electrostatic interactions, and self-assembly techniques, all of which help preserve the stability and efficacy of AMPs (77).

Moreover, combining AMPs with gold nanoparticles is highlighted as a promising approach to combat drug-resistant pathogens. Recent developments in the synthesis and antimicrobial activity of AMP-AuNPs were also discussed (77). While AuNPs show great potential as drug delivery carriers, their successful penetration through the intestinal barrier requires careful consideration of factors such as size, surface charge, rigidity, and shape (74). By optimizing the shape and surface chemistry of AuNPs, researchers aim to achieve better penetration and higher bioavailability through oral administration (77).

One of the major challenges in developing oral formulations of gold nanoparticles (AuNPs) is ensuring their stability as they pass through different environments and multiple biological barriers before reaching circulation. When AuNPs come into contact with the intestinal mucosa, their stability and the integrity of the encapsulated drug can be compromised. To address this, strategies such as using inhibitors and controlling tight junctions for paracellular transport have been proposed (77). Additionally, transcellular pathways involving endocytosis play a critical role in the cellular uptake of AuNPs. Clathrin-mediated uptake, a specific receptor-ligand interaction, has been extensively studied and is known to occur through non-specific endocytosis (77). The effectiveness of cellular internalization is strongly influenced by the physicochemical properties of AuNPs, such as size, surface charge, and shape (78).

For successful oral drug delivery using AuNPs, enhancing gastrointestinal absorption is crucial. One strategy involves attaching target receptors to the surface of the intestines and guiding the carrier towards these receptors, a process known as target receptor-mediated cellular internalization. To optimize this approach, nanocarriers are functionalized with ligands that specifically target receptors on specialized intestinal cells (78). When developing AuNPs for oral use, it is essential to consider factors like size, surface charge, rigidity, and shape. Various methods have been proposed to overcome the challenges of AuNPs penetrating the small intestine, thereby broadening their potential for oral applications (78).

Traditional iodinated contrast agents used in CT scans are recognized as promising alternatives when paired with gold nanoparticles (AuNPs). These agents offer advantages such as extended blood circulation time, the potential for disease-specific imaging, and enhanced contrast (79). The study also explored additional imaging techniques, including fluorescence imaging and photoacoustic imaging (PAI), revealing that AuNPs with tunable plasmonic absorption can achieve greater imaging depths compared to other optical methods used in PAI (79).

In fluorescence imaging, AuNPs can be functionalized with fluorescent dyes, which enhances their ability to produce high-resolution images of biological tissues (79). This method capitalizes on the biocompatibility and unique optical properties of AuNPs, enabling precise targeting and visualization of cellular and molecular processes (79).

Photoacoustic imaging (PAI) takes advantage of the tunable plasmonic absorption of AuNPs, allowing for imaging at greater depths than conventional optical methods. This technique is particularly valuable for detecting and monitoring deep-seated tumors and other challenging-to-image pathologies. By tuning the plasmonic absorption, AuNPs can be engineered to absorb specific wavelengths of light, which, upon excitation, generate acoustic waves that produce detailed images. This not only increases imaging depth but also enhances contrast and resolution, offering more precise diagnostic capabilities.

Additionally, the study discusses the potential of AuNPs in magnetic resonance imaging (MRI). When functionalized with specific ligands, AuNPs can target particular tissues or molecular markers, thereby improving the contrast and specificity of MRI scans. This targeted approach not only aids in the accurate diagnosis of diseases but also shows promise in real-time monitoring of therapeutic responses (79).

The integration of AuNPs into various imaging modalities underscores their versatility and significant potential in advancing molecular imaging. These innovations are set to enhance diagnostic accuracy, enable early disease detection, and improve the monitoring of therapeutic interventions (79).

Gold nanoparticles (AuNPs) have proven to be highly effective in applications related to food safety and health monitoring. Researchers have emphasized the importance of optical nanosensors in detecting food-related diseases, positioning AuNPs as a promising tool in this domain (80). They noted that traditional analytical methods are becoming increasingly inadequate, and that optical nanosensing, particularly with AuNPs, offers a superior alternative (80). This is due to the exceptional biocompatibility and high surface-to-volume ratio of AuNPs, which allow for the attachment of various organic and biological ligands through chemical and physical approaches (80).

The study also delves into the optical properties of AuNPs, exploring various types of optical sensing mechanisms used in AuNP-based nanosensors. It highlights effective sensing strategies that enhance the performance of these sensors. Additionally, the researchers conducted a comprehensive review of recent advancements in the use of AuNP-based optical nanosensors for monitoring pathogens, disease biomarkers, and food contaminants. They discussed the significant contributions of AuNP-based sensors in various analytical fields and their progress in recent years, addressing both the trends and challenges in the application of these nanosensors. The analysis provides a detailed overview of sensor mechanisms and recent developments in optical nanosensors, underscoring the potential of AuNPs to revolutionize food safety and health monitoring (80).

As gold nanoparticles (AuNPs) continue to show promise in food safety and health monitoring, their potential in the medical field is further enhanced by the rise of computational pharmaceutics. This new discipline, which combines artificial intelligence (AI) and big data, aims to improve drug delivery systems using advanced modeling techniques. By analyzing large datasets, AI algorithms and machine learning tools can predict how drugs will behave, optimizing the design and application of AuNPs in treatment strategies (80).

AI technologies, such as automated workflows, databases, and artificial neural networks (ANNs), are transforming drug discovery. They enable sophisticated data analysis, predict disease progression, and evaluate the pharmacological profiles of drugs, making the development of AuNP-based therapies more efficient (81, 82). For example, Target Fishing (TF) methods, which utilize AI and machine learning, speed up the identification of biological targets, enhancing both drug development and delivery systems. This integration represents a major advancement in pharmaceutical research and development, especially in optimizing AuNPs for targeted drug delivery (82).

In modeling the biological characteristics of nanomaterials like AuNPs, several steps are involved. Initially, nanomaterials are created and tested in biological experiments to gather data for training machine learning models. These models use descriptors of the physicochemical properties of nanomaterials to predict important attributes (83). Validation of these models is done through methods such as cross-validation and predicting outcomes for materials not used in model creation.

Machine learning plays a crucial role in processing large datasets and identifying complex patterns, which is especially important in precision cancer therapy. When combined with nanotechnology, AI can revolutionize various systems, including tumor identification, treatment decision-making, and the optimization of nanomedicine formulations (84). Specifically, in the context of AuNPs, AI can help predict their interactions with drugs, biological media, and cell membranes, as well as improve drug encapsulation and release kinetics (82).

By integrating these computational techniques, the development and application of AuNPs in medicine can be significantly advanced, leading to more effective and precise treatments.

Pihlajamäki et al. (85) utilized AI and machine learning (ML) to develop a dynamic model that approximates the 3-dimensional (3D) appearance of gold nanoparticles (AuNPs). These foundational concepts are shaping the future of precision cancer therapy and patient treatment planning, especially when integrating nanotechnology with AI. By combining mathematical modeling with AI, researchers are enhancing our understanding of nanoparticle efficiency (85, 86). AuNPs are particularly valuable in drug delivery research due to their unique properties, including stability, ease of synthesis, and the ability to produce various sizes. They can be loaded with small molecules, peptides, proteins, and nucleic acids in different therapeutic configurations, either covalently or non-covalently attached to the nanoparticles (86).

Several studies have employed computational methods to investigate the attributes of AuNPs and their interactions with various molecules. For instance, Bahareh Khodashenas and colleagues (87) conducted both experimental and computational studies on bovine serum albumin (BSA)-conjugated gold nanoparticles as a drug delivery system for curcumin. They synthesized AuNPs in three sizes (20 nm, 50 nm, 100 nm) and conjugated them with BSA, with and without 1,4 Dithiothreitol (DTT). The study found that larger AuNPs exhibited higher encapsulation efficiency for curcumin than smaller ones. Additionally, nanoparticles conjugated with both BSA and DTT demonstrated superior encapsulation efficiency compared to those with BSA alone. Curcumin release was more pronounced in acidic conditions, with the release rate varying based on nanoparticle size. The research also evaluated the antibacterial activity of the curcumin-loaded nanoparticles, demonstrating enhanced effectiveness compared to free curcumin (88).

In another study, Zhoumeng Lin et al. (87) developed a deep learning neural network model using physiologically based pharmacokinetic (PBPK) modeling to predict the delivery efficiency of nanoparticles to tumors based on their physicochemical properties, tumor models, and cancer types. The model showed superior accuracy over other machine learning methods, emphasizing the importance of properties like zeta potential and core material in influencing delivery effectiveness. This study highlights the integration of AI with PBPK modeling as a critical advancement in cancer nanomedicine, underscoring its potential for enhancing nanoparticle-based cancer therapies through precise predictive modeling and personalized treatment strategies (87).

Additionally, Lee and Atterberg (87) studied the effects of conjugating AuNPs on the structure and conformational flexibility of peptides. They investigated six peptides in water, both free and conjugated to AuNPs, and found that conjugation had a sequence-dependent effect on the peptides' dynamics and structure. For peptides with minimal secondary structure, adsorption onto the AuNP surface could lead to the loss of specific interactions between cellular constituents. This finding suggests that peptides with significant secondary structures in solution may be more suitable for peptide-nanoparticle conjugation in drug delivery applications (89).

These interconnected studies underscore the significant advancements in integrating computational methods, AI, and nanotechnology to optimize AuNPs for various biomedical applications, including drug delivery, cancer therapy, and the design of nanoparticle-peptide conjugates.

## 7 Limitations and challenges

While computational modeling offers valuable insights into the interactions between gold nanoparticles (AuNPs) and pharmaceutical molecules, it is essential to acknowledge the inherent limitations of this approach. One of the primary challenges is accurately capturing the complexity of biological systems in computational models. The behavior of AuNPs in the intricate and dynamic *in vivo* environment can be influenced by numerous factors, including the presence of various biomolecules, the local microenvironment, and the body's immune response. For instance, orally administering AuNPs presents significant challenges due to the need for these nanoparticles to remain stable across diverse environments and traverse multiple biological barriers before entering systemic circulation (78). Interaction with the intestinal mucosa can disrupt the stability of AuNPs and the encapsulated drug, necessitating the use of inhibitors and techniques to control tight junctions for paracellular transport, as well as leveraging transcellular pathways like endocytosis (78).

The application of AuNPs in cancer treatment also presents several challenges that must be addressed to ensure their effectiveness and safety. One major concern is toxicity and biocompatibility. Although AuNPs are generally considered to have low toxicity, further research is needed to determine their long-term biocompatibility and potential cytotoxic effects, as unexpected toxic reactions may arise from their interactions with biological systems (90). Targeting efficiency is another significant hurdle, as precisely targeting cancer cells with AuNPs remains difficult. The enhanced permeability and retention (EPR) effect, which facilitates the accumulation of nanoparticles in tumor tissues, is often irregular and varies depending on the patient and tumor type (90).

Additionally, drug loading and release present challenges for the therapeutic effectiveness of AuNPs. Ensuring controlled delivery of the medication to the target site can be problematic, with premature drug release potentially increasing systemic toxicity and reducing therapeutic efficacy. The interactions between AuNPs and the immune system can also fluctuate; while they may enhance immune responses against cancer, they could also provoke unfavorable immune reactions, leading to immunosuppression or inflammation (90).

Scalability and reproducibility are crucial factors in the large-scale synthesis of AuNPs, where consistent quality, size, and functionalization are necessary for their efficacy and safety (90). Variations in these factors can significantly impact the outcomes of nanoparticle-based treatments. Additionally, regulatory and manufacturing challenges must be addressed, as extensive research is required to demonstrate the safety and efficacy of AuNP-based medications for regulatory approval. Manufacturing these products to comply with stringent quality control standards can be both costly and time-consuming (90).

In the realm of computational challenges, one of the primary issues in cancer research is data integration and interpretation. Combining various genomic data types, such as DNA sequencing, RNA sequencing, and epigenetic data, is crucial for obtaining a comprehensive understanding of the cancer genome (91). However, this integration requires advanced computational methods and significant resources to manage and analyze the vast amounts of data involved. Clinical translation is another significant challenge, where computational findings must be validated and translated into clinical practice (91). This process involves validating predicted driver mutations and developing targeted therapies, requiring close collaboration between computational biologists, clinicians, and pharmaceutical researchers to ensure these findings can be effectively and safely applied in a clinical setting.

Accurately identifying structural variants (SVs) presents additional challenges due to limitations in current sequencing technologies and computational algorithms, often resulting in false positives and negatives (92). The complexity and diversity of SVs, including large insertions, deletions, duplications, inversions, and translocations, necessitate advanced computational methods for precise detection and interpretation (92). Integrating data from different sequencing technologies and genomic data types is essential for comprehensive SV detection but poses a significant challenge (92). Furthermore, determining the clinical relevance of detected SVs for precision oncology is complex and requires linking these variants to specific cancer types, patient outcomes, and therapeutic responses. Developing scalable and reproducible computational methods capable of handling large-scale genomic data and providing consistent results is critical for clinical application (92).

Future research should focus on understanding *in vivo* factors that affect the accuracy of computational methods used in studying AuNPs. Another limitation of computational modeling is the incomplete understanding of the underlying mechanisms governing the interactions between AuNPs and pharmaceutical molecules. While significant progress has been made, many unanswered questions remain, particularly regarding the impact of nanoparticle physicochemical properties on their biological performance. Overcoming these challenges requires large datasets, stronger collaboration between experts in nanomaterials, medicine, and computer science, and the integration of computational approaches at all stages of academic and industrial research to optimize their performance and clinical relevance.
